# Hair Growth Promoting Effects of Solubilized Sturgeon Oil and Its Correlation with the Gut Microbiome

**DOI:** 10.3390/ph17091112

**Published:** 2024-08-23

**Authors:** Jihee Kim, Jinho An, Yong-kwang Lee, Gwangsu Ha, Hamin Ban, Hyunseok Kong, Heetae Lee, Youngcheon Song, Chong-kil Lee, Sang Bum Kim, Kyungjae Kim

**Affiliations:** 1 Department of Pharmacy, College of Pharmacy, Sahmyook University, Seoul 01795, Republic of Korea; geeheeangel@naver.com (J.K.); romang1230@naver.com (J.A.); hskong0813@gmail.com (H.K.); hite486@gmail.com (H.L.); alexsongsu@syu.ac.kr (Y.S.); 2 Sturgeon Bio Co., Ltd., Cheongju 28581, Republic of Korea; lyg@sturgeonbio.com; 3 Department of Animal Life Resources, College of Science and Technology, Sahmyook University, Seoul 01795, Republic of Korea; hasu0058@gmail.com; 4 Institute for Artificial Intelligence and Biomedical Research, Medicinal Bioconvergence Research Center, College of Pharmacy, Yonsei University, Incheon 21983, Republic of Korea; hmban@target.re.kr; 5 Department of Manufacturing Pharmacy, College of Pharmacy, Chungbuk National University, Cheongju 28160, Republic of Korea; cklee@cbnu.ac.kr

**Keywords:** solubilized sturgeon oil, gut microbiota, androgenetic alopecia, hair growth

## Abstract

Androgenetic alopecia is a common disease that occurs in both men and women. Several approved medications have been used to treat this condition, but they are associated with certain side effects. Therefore, use of extracts derived from natural products, such as Siberian sturgeon (*Acipenser baerii*), and the regulation of the gut microbiota have become important topics of research. Sturgeon is known for its high nutritional value and anti-inflammatory properties; however, its effects on androgenetic alopecia and gut microbiota remain uncharacterized. Here, we aimed to investigate whether solubilized sturgeon oil (SSO) promotes hair growth and regulates the gut microbiome. C57BL/6 mice were divided into four groups. Three groups received topical applications of distilled water, SSO, or minoxidil, and one group was orally administered SSO. Each treatment was administered over 4 weeks. Histopathological analysis revealed a significant increase in follicle number (*p* < 0.001) and follicle diameter (*p* < 0.05). Immunohistochemical analysis revealed upregulation of β-catenin and ERK-1, markers involved in hair growth-promoting pathways. Furthermore, microbiome analysis revealed that the reduced gut microbiota was negatively correlated with these markers. Our findings indicate that oral administration of SSO promotes hair growth and regulates the abundance of hair growth-promoting gut microbiota.

## 1. Introduction

Gut microbiota refers to the community of microorganisms, including bacteria, viruses, protozoa, and fungi, that are present in the intestinal system [1]. The gut microbiome is influenced by dietary habits, and long-term dietary habits continuously affect the ecological formation of the gut microbiota [2]. In addition, the gut microbiota is crucial for maintaining human health, as it is associated with various diseases, including cardiovascular disease, cancer, and diabetes. Therefore, regulation of the gut microbiota is a key strategy in disease therapy [3].

Androgenetic alopecia is hair loss that affects both men and women, but it is more common in men [4]. Hair growth involves the anagen (growth phase), catagen (regression phase), and telogen (resting phase) cycles in hair follicles [5]. Androgenetic alopecia is caused by the shortening of the growth stage or prolongation of the regression and resting stages [6]. Many signal molecules and growth factors are associated with hair cycle and hair follicle proliferation. Insulin-like growth factor-1 (IGF-1) participates in the initiation of the anagen stage [7,8]. Transforming growth factor beta (TGF-β) inhibits the growth and differentiation of hair follicles [9]. In addition, epidermal growth factor (EGF) increases the levels of extracellular signal-regulated kinase 1/2 (ERK-1/2) with hair follicle-derived mesenchymal stem cell proliferation [10], and activation of p38 by hepatocyte growth factor (HGF) induces the proliferation of melanoma cells [11]. The drug approved by the Food and Drug Administration (FDA), minoxidil, has been used to treat hair loss [12]. Minoxidil promotes hair growth by inducing β-catenin activity, stimulating the growth, proliferation, and differentiation of hair follicles, upregulating VEGF, and maintaining hair growth. However, this drug has serious side effects, such as weight gain, edema, angina pectoris, ischemic heart disease, and pulmonary hypertension [13,14]. Therefore, the development of a hair loss prevention drug that has fewer side effects during long-term use is crucial.

The composition of the gut microbiota is affected by diet. There have been reports of decreased gut microbiome diversity in mice fed a high-fat diet [15]. In another study, a high-fat diet changed the ratio of the abundance of *Ruminococcaceae* and *Rikenellaceae* [16]. The effect of diet on human intestinal microbial communities in humanized mice has also been reported [17]. Furthermore, changes in the gut microbiota are linked to androgenetic alopecia. In a study, the intestinal microbiota of patients suffering from alopecia areata was identified. The major gut microbes in patients with alopecia areata were found to belong to the *Lachnoclostridium*, *Bifidobacterium*, *Streptococcus*, and *Eubacterium* genera, and *Firmicutes*, *Proteobacteria*, and *Actinobacteria* phyla. In particular, *Firmicutes* and *Proteobacteria* were highly abundant, indicating that the patient’s microbiota was disturbed [18]. Previous studies have shown differential abundance of the gut microbiome in women and men. In women, the abundance of *Lachnoclostridium, Oscillospiraceae NK4A214 group*, and *Escherichia/Shigella* was higher and that of *Bifidobacterium* was lower in androgenetic alopecia patients than in healthy controls. In men, the abundance of *Butyricicoccus*, *Ruminococcaceae incertae sedis*, *Parabacteroides*, *Christensenellaceae R7 group*, *Alistipes*, and *unclassified oscillospiraceae* was lower in androgenetic alopecia patients than in the healthy controls [19]. Oral administration of *Akkermansia muciniphila* mediates hair regrowth against testosterone-induced hair growth inhibition [20]. Moreover, increased abundance of intestinal *Lactobacillus murinus* induced alopecia in C57BL/6 mice deficient in biotin, a major component in hair formation. Alopecia symptoms occurred in mice fed a biotin-deficient diet. These mice were monocolonized with *Lactobacillus murinus*, which apparently resulted in more severe alopecia symptoms compared with that in the control group. The authors surmised that alopecia was aggravated because excessive *Lactobacillus murinus* consumed the remaining biotin [21]. Based on the abovementioned reports, regulating the gut microbiota with diet may be an effective strategy for preventing hair loss.

The Siberian sturgeon (*Acipenser baerii*) is a freshwater fish that is distributed along Siberian rivers such as the Ob, Yenisei, Lena and Kolyma rivers [22]. In general, *Acipenser* sturgeon and caviar are known for their high nutritional value, particularly their docosahexaenoic acid (DHA) and eicosapentaenoic acid (EPA) content [23]. DHA, an omega-3 fatty acid, induces hair growth by stimulating the growth pathway of dermal papilla cells (DPCs) [24]. A deficiency in omega-3 may induce alopecia by increasing the proportion of follicles in the telogen phase [25]. Fatty acids play a significant role in alopecia. In women, supplementing omega-3 and omega-6 fatty acids with antioxidants for six months resulted in improved hair density, a reduction in the telogen ratio, and an increase in the proportion of anagen hairs [26]. Additionally, when rhesus macaques were supplemented with fatty acids, hair loss improved, suggesting that fatty acid supplementation could be a strategy for treatment of hair loss in primates [27]. The omega-6 polyunsaturated fatty acid, arachidonic acid, can promote hair growth by increasing the expression of growth factors, such as FGF-7, FGF-10, and HGF. In a mouse experiment, topical application of arachidonic acid to shaved areas resulted in elongation of the anagen phase of the hair cycle [28]. This research suggested that owing to its high fatty acid content, dietary treatment with sturgeon extract can be expected to sufficiently promote hair growth through the activation of DPCs. The beneficial effects of consuming omega-3 fatty acids on the gut have been extensively investigated [29,30,31].

Direct administration of Japanese sturgeon (*Acipenser schrenckii*) hydrolysates was reported to regulate the changes in the gut microbiota of mice and alleviated dextran sulfate sodium-induced colitis [32]. These results indicate that sturgeon constituents may positively affect the gut microbiota. Taken together, these two findings indicate that dietary treatment with sturgeon constituents can potentially activate DPCs for hair growth and can bring about positive changes in the microbiota conducive to hair growth.

In this study, we investigated the effects of solubilized sturgeon oil (SSO) on hair growth in human follicle DPCs (HFDPCs) and C57BL/6 mice. SSO is an oil-in-water preparation of sturgeon oil isolated from boiling water extracts of the whole body of Siberian sturgeon, which was reported to exert therapeutic effects on house dust mite-induced atopic dermatitis in mice [33]. We have shown here that SSO also exerts hair growth-promoting effects. Additionally, we examined the relationship between SSO-induced hair growth and changes in the composition of the mouse gut microbiota. Our results show that SSO promotes hair growth by inducing the proliferation of DPCs and altering gut microbiota composition.

## 2. Results

### 2.1. Treatment with SSO Promotes Hair Growth in Mice

**Figure 1:**
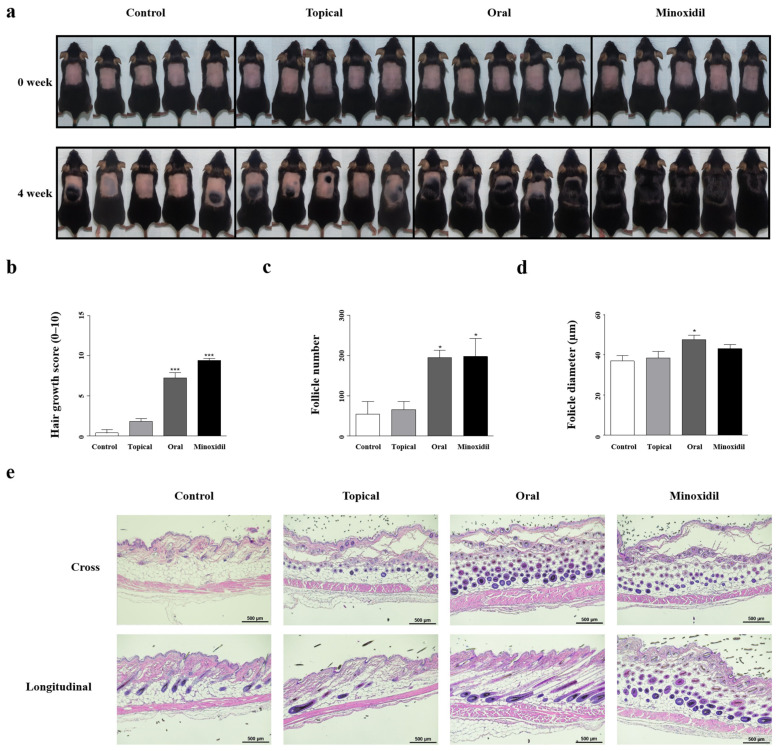
SSO treatment promotes hair growth in mice. (**a**) The treatments were as follows: Control, mice treated topically with distilled water-containing gel; Topical, mice treated topically with SSO-containing gel; Oral, mice fed SSO orally; Minoxidil, mice treated topically with minoxidil. Photographs were captured weekly after shaving (pink indicates the telogen phase and black indicates the anagen phase). (**b**) Hair growth area was measured using ImageJ based on the shaved area at week 0. The scores represent the percentage of hair growth as follows: 0, 0–9% (no hair); 1, 10–19%; 2, 20–29%; 3, 30–39%; 4, 40–49%; 5, 50–59%; 6, 60–69%; 7, 70–79%; 8, 80–89%; 9, 90–99%; and 10, 100%. (**c**,**d**) Follicle number and diameter were measured using cross and longitudinal sections, respectively. (**e**) Formalin-fixed mice dorsal skin stained with hematoxylin and eosin. Data are presented as the mean ± standard deviation (SD) (N = 5 in each group). Statistical significance is denoted as * *p* < 0.05 and *** *p* < 0.001.

We investigated the hair growth-promoting effects of SSO in shaved C57BL/6 mice. Mice in the SSO-oral treatment group and the minoxidil treatment group had pink skin in the telogen phase and dark skin when transitioning to the anagen phase, the same phenomenon described in a previous paper [34], with skin turning gray starting at week 3 (Figure 1a). This indicated a change from the telogen phase to the anagen phase because of the treatment. The extent of hair growth, 4 weeks after the start of the experiment, was analyzed and scored from 0 to 10 to evaluate the degree of hair growth (Figure 1b). The hair growth promotion scores of the SSO-oral treatment group and minoxidil treatment group were significantly (*p* < 0.001) higher than those of the negative control group (distilled water-gel treatment) and SSO-topical treatment group, increasing by 1650% and 2400%, respectively. Therefore, the SSO-oral treatment group showed a hair growth-promoting effect equivalent to that in the minoxidil group, whereas no significant hair growth-promoting effect was observed in the SSO-topical treatment group.

Histopathological observations and evaluations of follicle quantity and diameter were performed. Follicles were counted using cross-sections of the tissue, and follicle diameter was measured using longitudinal sections (Figure 1c–e). The number and diameter of follicles in the oral group were significantly (*p* < 0.05) higher than those in the control group, increasing by 257.2% and 246.6%, respectively. The ineffective results for topical treatment indicate that only small quantities of SSO might have reached DPCs by permeating through the epidermis. These results demonstrate that oral administration of SSO increases the number and diameter of follicles and promotes hair growth. Thus, dietary intake of SSO likely allows the delivery of functional substances to DPCs.

### 2.2. Treatment with SSO Induces Changes in Growth Factor Expression

**Figure 2:**
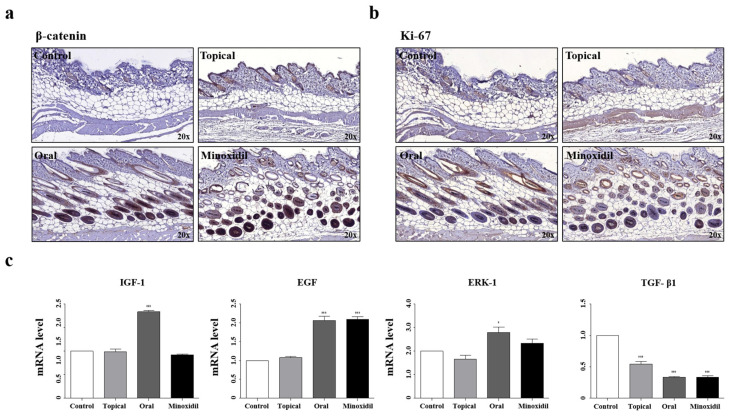
Treatment with SSO induces changes in the expression of growth factors. (**a**,**b**) Representative immunohistochemical images of the dorsal skin of mice. The skin was stained for β-catenin and Ki-67. (**c**) Total RNA was obtained from mice dorsal skin, and the relative mRNA level of each growth factor was determined via qRT-PCR. Data are presented as the mean ± SD (N = 5). Statistical significance is denoted as * *p* < 0.05 and *** *p* < 0.001.

Our findings revealed that oral administration of SSO promotes hair growth and hair growth-promoting gut microbiota (Figure 2a,b). Immunohistochemical analysis was conducted to detect the expression of the hair growth markers β-catenin and Ki-67. Both markers showed higher expression in the oral and minoxidil groups than in the control group. In contrast, the topical and control groups did not exhibit any marker expression.

We confirmed mRNA levels of hair growth-related genes in the mouse dorsal skin using qRT-PCR (Figure 2c). It has been demonstrated in many previous studies that an increase in the growth factor promotes hair growth [7,8,9,10,11]. The oral group showed a significant increase in IGF-1, EGF, and ERK-1 levels (*p* < 0.001 and *p* < 0.05, respectively). Notably, the levels of TGF-β1 decreased in the topical, oral, and minoxidil groups (*p* < 0.001). These results indicate that oral administration of SSO affects hair growth by regulating growth factors.

### 2.3. SSO Promotes the Proliferation of HFDPCs

**Figure 3:**
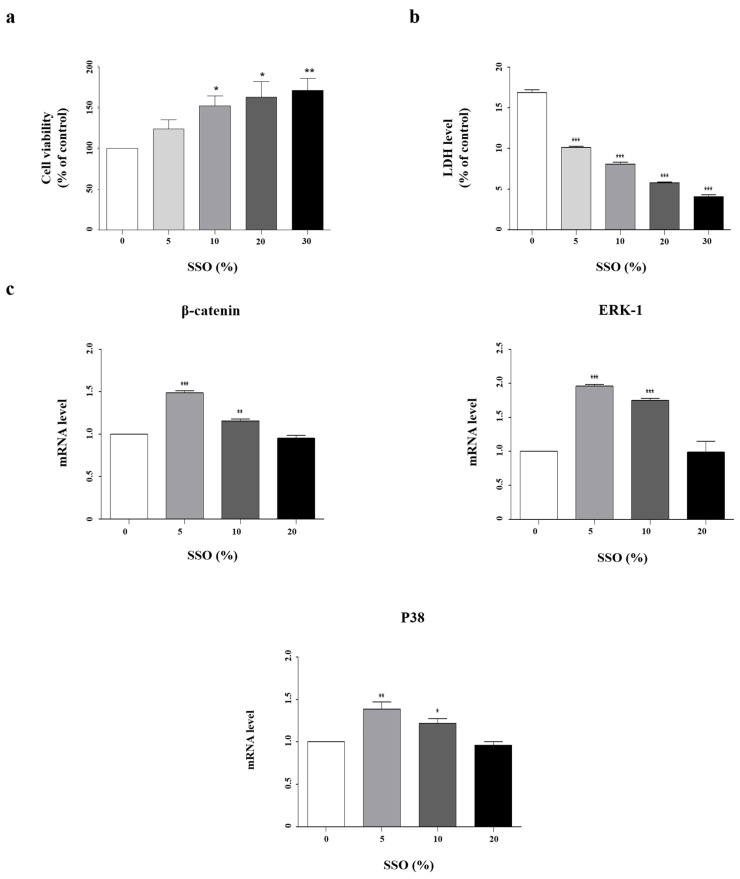
Treatment with SSO promotes the proliferation of human follicle dermal papilla cell (HFDPCs). (**a**,**b**) Effect of SSO on HFDPCs. (**c**) Total RNA was purified from HFDPCs, and the relative mRNA level of each growth factor was determined via qRT-PCR. Data are presented as the mean ± SD (N = 5). Statistical significance is denoted as * *p* < 0.05, ** *p* < 0.01, and *** *p* < 0.001.

DPCs play an important role in hair follicle proliferation, differentiation, and cycle control. DHT, which causes hair loss, also causes DP miniaturization, resulting in alopecia [35,36]. Cell viability was measured after treatment with SSO at concentrations of 5, 10, 20, and 30% to assess its effect on HFDPCs. Cell viability significantly (*p* < 0.05, *p* < 0.05, and *p* < 0.01, respectively) increased at 10, 20, and 30%, and LDH levels decreased (*p* < 0.001) in a dose-dependent manner (Figure 3a,b).

Additionally, we conducted qRT-PCR after treating HFDPCs with SSO to confirm changes in the mRNA levels of growth signaling pathway (Figure 3c). β-catenin, ERK-1, and p38 levels were significantly increased at 5 and 10% (*p* < 0.001, *p* < 0.01, and *p* < 0.05). These findings suggest that SSO promotes the proliferation of HFDPCs without cytotoxicity and may promote hair growth.

### 2.4. Oral Administration of SSO Alters Gut Microbiota Composition

Previous studies have shown that the composition of the gut microbiota is affected by diet and that hair growth and gut microbiota are correlated [15,16,17,18]. In the present study, we confirmed the effect of oral administration of SSO on the gut microbiota and analyzed its correlation with hair growth.

**Figure 4:**
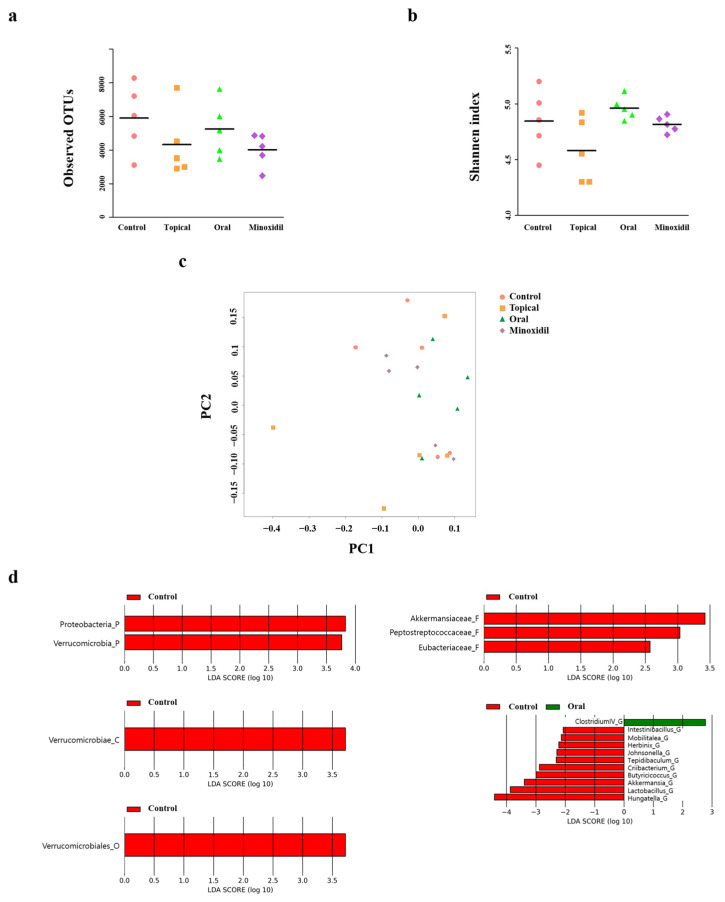
Oral administration of SSO alters the gut microbiota composition. (**a**,**b**) Alpha (α)-diversity was analyzed by measuring the observed OTUs at the genus level, and the Shannon index was used to assess evenness and richness within groups. (**c**) Beta (β)-diversity was assessed using principal coordinate analysis (PCoA) to measure the similarity of microbial diversity among the five groups. (**d**) Linear discriminant analysis effect size (LEfSe) was conducted based on the results of 16S rRNA sequencing performed on DNA extracted from the colon of each mouse. The bar graphs for each group indicate strains that had increased abundance compared to the control. Each dataset was analyzed using the Kruskal–Wallis test followed by the Wilcoxon test. Data are presented as the mean ± SD (N = 5).

Microbial analysis of the cecal contents in each group was performed using 16S rRNA sequencing. Alpha (α)-diversity analysis was conducted to assess the diversity of species within each sample, using operational taxonomic units (OTUs) and the Shannon index to represent species evenness and richness, respectively. The OTU values for the control, topical, oral, and minoxidil groups were 5900.4, 5253, 4907.6, and 4017.4, respectively. The corresponding Shannon index values were 4.84632, 4.96209, 4.878258, and 4.817286, respectively. The OTU values tended to be lower in the control group than in the minoxidil group, indicating lower microbial species diversity. No significant differences in the Shannon index values were found among the groups (Figure 4a,b). Beta (β)-diversity was analyzed using principal coordinate analysis (PCoA) to evaluate microbial species diversity between groups. No distinct differences in similarity were observed between the groups (Figure 4c). These results suggest that SSO had a minimal impact on the species diversity of the gut microbiota in mice in the oral and topical groups.

Oral administration of SSO did not significantly affect the diversity of the gut microbiota; therefore, we conducted a LEfSe to investigate its influence on the composition of gut microbial communities (Figure 4d). In the control group, the phyla *Proteobacteria* and *Verrucomicrobia*, the class *Verrucomicrobiae*, the order *Verrucomicrobiales*, the families *Akkermansiaceae*, *Peptostreptococcaceae*, and *Eubacteriaceae*, and the genera *Intestinibacillus*, *Mobilitalea*, *Herbinx*, *Johnsonella*, *Tepidibaculum*, *Criibacterium*, *Butyricicoccus*, *Akkermansia*, *Lactobacillus*, and *Hungatella* emerged as representative gut microbiota. The genus *Clostridium IV* was representative of the gut microbiota in the oral group when compared with that in the control group. These results indicate that oral administration of the SSO changes the composition of the gut microbiota.

### 2.5. Correlation between Selected Gut Microbiota and the Number and Diameter of Hair Follicles

**Figure 5:**
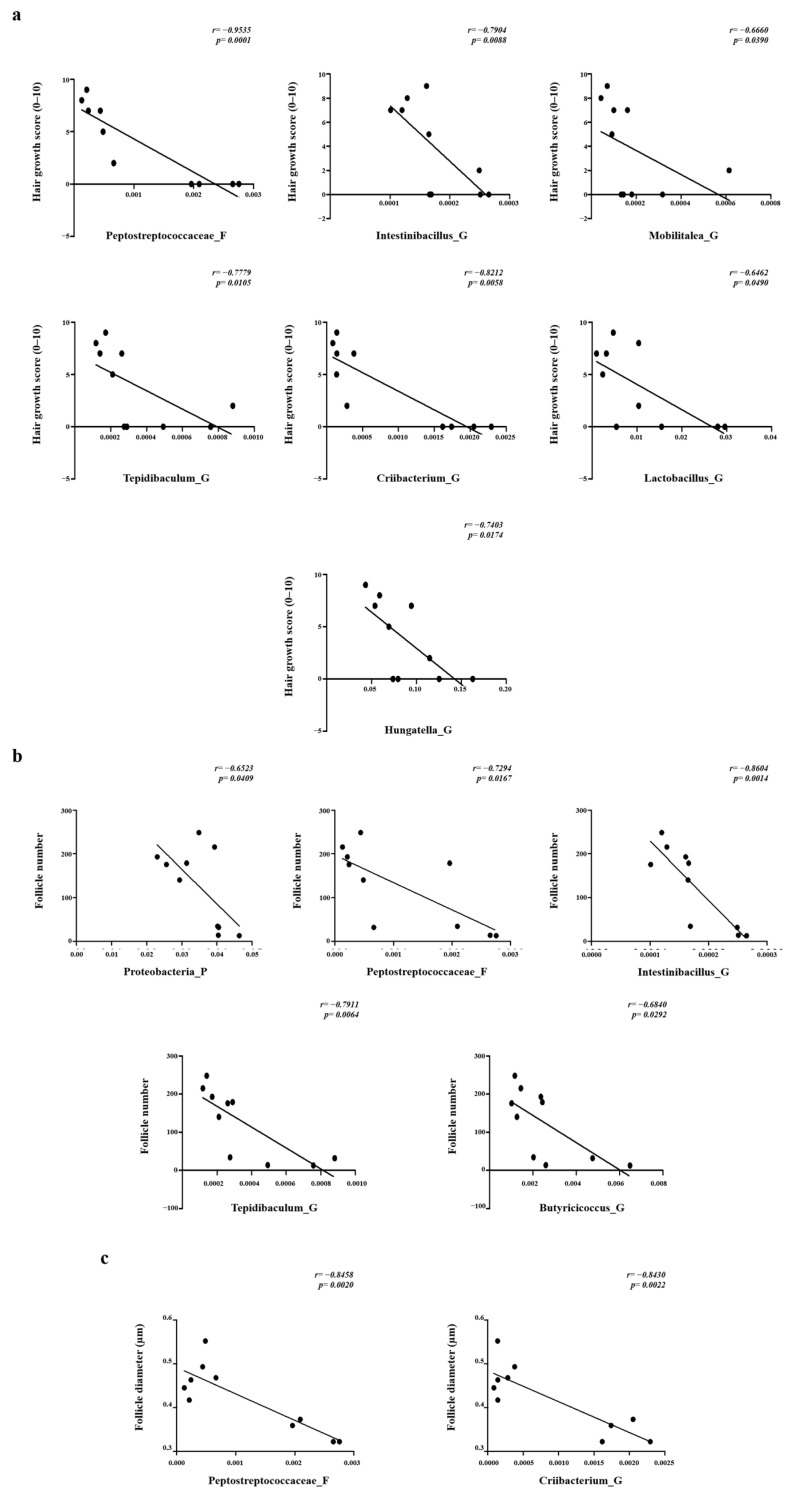
Correlation between gut microbiota communities and the (**b**) number and (**c**) diameter of hair follicles. Correlations were visualized through clustering using Spearman’s rank correlation analysis. Data are presented as the mean ± SD (N = 5).

The correlations of the selected gut microbiota with the hair growth score and the number and diameter of hair follicles were analyzed. The hair growth score was negatively correlated with the family *Peptostreptococcaceae* (*p* < 0.001) and genera *Intestinibacillus* (*p* < 0.01), *Mobilitalea* (*p* < 0.05), *Tepidibaculum* (*p* < 0.05), *Criibacterium* (*p* < 0.01), *Lactobacillus* (*p* < 0.05), and *Hungatella* (*p* < 0.05). The number of hair follicles was negatively correlated with the phylum *Proteobacteria* (*p* < 0.05), family *Peptostreptococcaceae* (*p* < 0.05), and genera *Intestinibacillus* (*p* < 0.01)*, Tepidibaculum* (*p* < 0.01), and *Butyricicoccus* (*p* < 0.05) (Figure 5a). Additionally, hair follicle diameter was negatively correlated with the family *Peptostreptococcaceae* (*p* < 0.01) and genus *Criibacterium* (*p* < 0.01) (Figure 5b). *Proteobacteria* constitutes an independent phylum, whereas *Peptostreptococcaceae*, *Intestinibacillus*, *Tepidibaculum*, *Butyricicoccus*, and *Criibacterium* belong to the order *Eubacteriales* within the phylum *Bacillota*. Among these, *Intestinibacillus,* which was the most significantly correlated, *Peptostreptococcaceae*, and *Criibacterium* represented approximately 0.02, 0.1, and 0.08% of the overall gut microbiota composition, respectively. The top 20 strains in each taxonomic group accounted for approximately 99.2% at the family level and 82% at the genus level. The results in Figure 4 and Figure 5 suggest that dietary treatment with SSO does not significantly alter the proportion of dominant microbes in the gut microbiota community, but does influence the composition of other microbial taxa.

## 3. Discussion

In this study, we confirmed that dietary intake of SSO leads to the activation of DPCs, increases the levels of growth factors in the skin tissue, and changes the microbiota. Furthermore, this approach enabled us to identify the microbiota that correlated negatively with hair growth.

We found that the oral intake of SSO was significantly more effective in promoting hair growth than topical treatment. The topical treatment of SSO showed negligible effects on hair growth. For growth hormone levels, such as IGF-1, EGF, and ERK-1 in the mice dorsal skin tissues, which received topical penetration of SSO, we observed no significant differences compared to the control group. These findings suggest that direct skin treatment of SSO does not exert a substantial impact on DPCs. By contrast, the oral intake of SSO leads to an increase in the level of growth hormones, such as IGF-1 and EGF, subsequently promoting hair growth. In particular, treatment of DPCs, which play a crucial role in the promotion of hair growth [37], with SSO, resulted in the upregulation of genes involved in differentiation and growth, such as β-catenin, ERK-1, and p-38. This upregulation was associated with increased cell viability and inhibition of cell apoptosis. These results highlight the potential of SSO in enhancing hair growth via DPC activation. Sturgeon oil, rich in omega-3 fatty acids such as DHA and EPA, has been suggested to have potential in promoting hair growth [23,24]. DHA stimulates DPCs, essential for hair follicle development, and a deficiency in omega-3 can lead to increased hair loss by shifting hair follicles to the telogen phase [25]. Studies have also demonstrated that supplementing omega-3 and omega-6 fatty acids, along with antioxidants, improves hair density and the proportion of anagen hairs [26]. Given its high fatty acid content, dietary treatment with sturgeon oil could be expected to promote hair growth through DPC activation. Our study underscores the potential of sturgeon oil as a natural therapeutic strategy for hair loss prevention and treatment. Further research on sturgeon oil on the activation of hair follicle stem cells (HFSCs), which significantly contribute to hair growth [38,39,40], may help us understand the mechanisms by which sturgeon oil promotes hair growth.

Another important discovery regarding the functionality of SSO is its impact on the gut microbiota. The regulation of various processes in the human body by the gut microbiota has been extensively researched and is well documented [18,19,41]. The role of the gut microbiota in promoting hair growth has been explored in previous studies. The finding that sturgeon extract maintains the overall balance of the gut microbiota while inhibiting harmful bacteria that may negatively affect hair growth sets our study apart from previous ones. Our results indicate that SSO selectively inhibits harmful bacteria, ensuring its safety.

The homeostasis of gut microbiota is a crucial factor for intestinal health. The changes in gut microbiota induced by the SSO used in this study were minimal. Notably, the genera *Intestinibacillus* and *Criibacterium*, and family *Peptostreptococcaceae*, were significantly decreased, accounting for 0.02, 0.2, and 0.08% of the overall gut microbiota community composition, respectively. This indicates that dietary consumption of SSO may influence small-scale gut microbiota communities without causing imbalances, highlighting its safety. Additionally, the bacterial strains reduced by SSO are known to increase during disease onset. *Peptostreptococcus* was found in low abundance in breastfed infants and *Peptostreptococcus anearobius* was abundant in patients with type 2 diabetes experiencing weight loss [42,43]. Studies have also shown that *Criibacterium bergonii* was isolated from vaginal samples from women with bacterial infections. Another study reports that the genus *Criibacterium* is abundant in postmenopausal endometrial cancer tissues [44,45]. *Intestinibacillus* sp. *Marseille-P4005* was shown to be positively correlated to the severeness of Coronavirus disease 2019 (COVID-19), and *Intestinibacillus massiliensis* has been reported as a dominant species in patients with colorectal cancer [46,47]. Most of these microorganisms are negatively correlated with COVID-19, cancer, and type 2 diabetes. Our study already confirmed that SSO decreases the number of *Intestinibacillus*, *Criibacterium*, *Peptostreptococcaceae* in the oral group. This suggests that the reduction in specific strains via treatment using SSO could potentially be used for disease control.

Further research confirming the inhibitory effect of SSO on harmful bacteria could demonstrate its efficacy under a wide range of conditions. Specifically, confirmation of the inhibition of harmful bacteria associated with diseases, such as cancer, chronic inflammatory bowel disease (IBD), and Crohn’s disease, would provide stronger evidence for the antibacterial properties of SSO.

Although our findings are important, this study had some limitations. Further research is required to accurately determine the correlation between hair growth-promoting effects and the gut microbiota. In addition, considering the fact that the gut microbial communities identified in this study are associated with the promotion of hair growth, we would need to determine the effects of microbial communities on the promotion or inhibition of hair growth and their underlying mechanisms.

## 4. Materials and Methods

### 4.1. Preparation of Solubilized Sturgeon Oil

The method for manufacturing SSO is described in detail in a patent registered with the Korea Intellectual Property Office [48], and the description of the core method used for manufacturing SSO, the analysis method and results for the total fatty acid content and composition contained in SSO are described in a previous report [33]. To summarize the main points again, the Siberian sturgeon was boiled, and the oil layer was separated and emulsified with purified water. This emulsion was combined with a herbal extract containing wild ginseng, *Camellia sinensis*, and *Chrysanthemum leucanthemum*, and aged for 10 days at 19–23 °C. The aged mixture was distilled to obtain SSO, which had a total lipid content of 0.53 ± 0.06%. The fatty acid composition was analyzed using gas chromatography–mass spectrometry.

SSO was used as is for oral administration, or was formulated into a semi-solid gel form by adding an equal ratio of carbomer (Lubrizol Korea Co., Ltd., Seoul, Republic of Korea) and L-arginine (Saesang Co., Seoul, Republic of Korea) for topical application to depilated skin. To prepare a gel, SSO was heated to 50 °C, 0.5% carbomer was added and stirred for 3 min using an immersion blender (SMX-D400FS, Shinil Electronics, Seoul, Republic of Korea) to dissolve, and then 0.5% arginine was added and stirred for an additional 5 min.

### 4.2. Chemicals

We purchased 3% and 5% minoxidil products from Hyundai Pharm Co., Ltd. (Gangnam-gu, Republic of Korea).

### 4.3. Cell Culture

HFDPCs were obtained from Promo Cell (C-12071; Heidelberg, Germany). They were cultured in Follicle Dermal Papilla Cell Growth Medium (ready-to-use; C-26501; Promo Cell) in a CO_2_-regulated incubator at 37 °C (Forma Series II 3111; Thermo Fisher Scientific, Waltham, MA, USA).

### 4.4. Cell Proliferation Assay

A cell counting kit (CCK-8; Dojindo Laboratories, Kumamoto, Japan) assay was conducted to confirm the proliferative effect of SSO on HFDPCs. HFDPCs were seeded in a 96-well plate at a density of 2 × 10^4^ cells/well/200 μL, with 5 wells per group. The cells were cultured in a CO_2_ incubator at 37 °C for 24 h. Then, the supernatant was removed, and the samples were treated with the 100 μL/well of SSO or serum-free media. The experimental groups were as follows: a control group, in which HFDPCs received no treatment (culture medium); an experimental group, in which cells were treated with SSO at 5, 10, 20, and 30% concentrations. The cells were then cultured for an additional 24 h at 37 °C in a 5% CO_2_ incubator. After incubation for 24 h, CCK-8 reagent was added to each well at a concentration of 10% and the absorbance was measured at 450 nm after 2 h.

### 4.5. Cytotoxicity Assay

A lactate dehydrogenase (LDH) assay kit (Cytotoxicity LDH Assay Kit-WST; Dojindo Laboratories, Kumamoto, Japan) was conducted to confirm the cytotoxicity of SSO on HFDPCs. HFDPCs were seeded in a 6-well plate at a density of 1 × 10^5^ cells/well/2 mL. The cells were cultured in a CO_2_ incubator at 37 °C for 24 h. Then, the supernatant was removed, and the plates were treated with the 1 mL/well of SSO or serum-free media. The experimental groups were as follows: a control group, in which HFDPCs received no treatment (culture medium); an experimental group, in which cells were treated with SSO at 5, 10, 20, and 30% concentrations. The cells were then cultured for an additional 24 h at 37 °C in a 5% CO_2_ incubator. The cells were then cultured for an additional 24 h at 37 °C in a 5% CO_2_ incubator.

Then, the supernatant was collected, and centrifuged at 1000 rpm for 5 min to remove all cells, aliquoted in a 96-well plate with 100 μL/well, and then LDH assay reagent was added at 100 μL/well. After incubating for 30 min, a stop buffer was added at 50 μL/well, and the absorbance was measured at 490 nm.

### 4.6. Evaluation of Hair Growth Promotion

C57BL/6 mice (6-weeks-old) were purchased from Samtako Bio Korea, Inc. (Osan, Republic of Korea). Animal experiments were approved by the Institutional Animal Care and Use Committee of Shamyook University (Seoul, Republic of Korea) [Approval Number: SYUIACUC 2024-002]. Female C57BL/6 mice (7-weeks-old) were anesthetized using isoflurane and then shaved on their dorsal surface. They were randomly separated into four groups (N = 4), named control, topical, oral, and minoxidil. Treatments were conducted over 4 weeks as follows: Control group, which was sprayed with purified water twice (approximately 500 μL) and then 1 mL of purified water-containing gel was applied to the back twice a day; Topical group, which was sprayed with SSO twice (approximately 500 μL) and then 1 mL of SSO-containing gel was applied to the back twice a day; Oral group, which has free consumption of SSO provided in a water bottle (average daily intake of 6 mL ± 0.3 mL); Minoxidil group, which had 1 mL of 1% minoxidil applied to the back twice a day. In previous studies, minoxidil was used at concentrations ranging from 1% to 5% [49,50,51,52]. In our study, we used 1% because the mice were treated with minoxidil twice a day.

### 4.7. Hematoxylin and Eosin (H&E) Staining

After completion of the mice experiments, the mice were euthanized using CO_2_ gas. The dorsal skin tissue was dissected from mice in each group and fixed in 10% neutral-buffered formalin. Vertical or horizontal sections (5 μm thick) of the dorsal skin tissue were sliced and fixed in paraffin. The sections were stained with hematoxylin and eosin (H&E). The stained areas were observed and photographed under a microscope (Olympus, Tokyo, Japan), and follicle diameter and follicle quantity were examined at 40× magnification.

### 4.8. ImageJ Analysis

Photographs of the dorsal skin of all mice were captured weekly using a camera from the initiation of treatment. Each individual was anesthetized with isoflurane, placed on graph paper, and photographed. Hair growth was assessed based on photographs captured between 0 and 4 weeks and analyzed using the ImageJ software version 1.8.0. Data are expressed as percentages and assigned scores based on the system described by Woo et al. (2019) [53]. Each assigned score was within the range of 0–10, where “0” indicates 0–9% hair loss, “1” indicates 10–19% hair growth, and so on, with “10” indicating 100% hair growth and a healthy condition.

### 4.9. Immunohistochemical Analysis

After completion of the mice experiments, the mice were euthanized using CO_2_ gas. The dorsal skin tissues were dissected from mice in each group and fixed in 10% neutral-buffered formalin. It was then cut into vertical or horizontal sections (5 μm-thick) and fixed in paraffin. The sections were deparaffinized and rehydrated. The samples were boiled in citrate buffer in a microwave (KR-B202W; Winia; Gwangju, Republic of Korea). After blocking in 2% goat serum in Tris-buffered saline with Triton X-100, the sections were incubated with the primary antibodies, mouse monoclonal anti-β-catenin (1:100; sc-7963; Santa Cruz Biotechnology, Dallas, TX, USA), mouse monoclonal anti-VEGF (1:100; sc-7269; Santa Cruz Biotechnology), and rabbit polyclonal anti-Ki-67 (1:200; ab15580; Abcam, Waltham, MA, USA). Envision+ System-HRP-labeled polymer anti-mouse antibody (K4000; Dako, Denmark) and anti-rabbit antibody (K4003; Dako, Denmark) were used for immunohistochemical analysis.

### 4.10. Microbiome Analysis

Total DNA was extracted from the mouse cecal contents using the Qiagen QIAamp^®^ DNA Stool Mini Kit (QIAGEN, Hilden, Germany). The cecal contents were obtained from the mouse cecum using pairs of forceps and scissors. Subsequently, the hypervariable region V3–V4 of the 16S rRNA was amplified using mixed primers, generating PCR products. The PCR products were then used to create libraries for DNA sequencing by attaching multiplexing indices and Illumina sequencing adapters, following the 16S Metagenomic Sequencing Library Preparation protocol of Illumina (San Diego, CA, USA). The final products, which included Illumina adapters and multiplexing indices, were approximately 630 bp in size. Subsequently, the MiSeq instrument was used for 2 × 300 bp paired-end sequencing, and demultiplexing based on barcodes was performed after the sequencing run.

Following MiSeq NGS, a preprocessing step was performed to remove Illumina adapter sequences and to filter out low-quality reads. This involved removing Illumina adapter sequences using Cutadapt, performing quality trimming with DADA2, and subsequently eliminating the phiX sequences. DADA2 was also used to calculate error rates, merge paired reads, and remove chimeric sequences. The generated amplicon sequence variant (ASV) table was used for taxonomic classification with the Silva database. Sparsity checks were performed to ensure an adequate number of reads for analysis, and sparsity curves were generated. Taxonomic classification at the levels of genus, family, order, class, phylum, and kingdom was conducted based on the sparsified ASV table, and the relative abundances were calculated. The obtained data were visualized using Krona plots.

Alpha (α)- and beta (β)-diversity analyses were conducted to assess diversity within each sample and differences in the diversity between samples, respectively. Statistical analysis of α-diversity indices was performed using nonparametric Wilcoxon rank-sum tests, and β-diversity was visualized using nonmetric multidimensional scaling plots based on Bray–Curtis dissimilarity, Jaccard distance, and UniFrac distance (weighted/unweighted).

A permutation multivariate analysis of variance (PERMANOVA) was applied to determine intergroup differences. A bias-corrected analysis of composition of microbiomes was conducted to identify the significant differences in bacterial abundance among the groups. Microbial community composition in the gut was normalized based on the calculated taxonomic richness, and α-diversity and β-diversity analyses were performed. Linear discriminant analysis effect size (LEfSe) was employed to estimate taxonomic richness and characterize differences between groups using the online Galaxy web platform tool.

### 4.11. Quantitative RT-PCR

HFDPCs were seeded in a 6-well plate at a density of 1 × 10^5^ cells/well/2 mL, with 1 well per group. The cells were cultured in a CO_2_ incubator at 37 °C for 24 h. Then, the supernatant was removed, and the plates were treated with the 1 mL/well of SSO or serum-free media. The experimental groups were as follows: a control group, in which HFDPCs received no treatment (culture medium); experimental groups, in which cells were treated with SSO at 5, 10, and 20%. The cells were then cultured for an additional 24 h at 37 °C in a 5% CO_2_ incubator. Then, the supernatant was discarded.

Total RNA of HFDPCs and dorsal skin tissues of mice was isolated using a PURY RNA PLUS kit (GenDEPOT, Katy, TX, USA) according to the manufacturer’s instructions. AccuPower RT PreMix (Bioneer, Daejeon, Republic of Korea) was used for cDNA synthesis. The quantitative real-time polymerase chain reaction (qPCR) was performed using QuantStudio^TM^ 3 (Applied Biosystems, Waltham, MA, USA) system with SYBR (SensiFast SYBR Lo-Rox Mix 2x, Meridian Bioscience, Cincinnati, OH, USA). The primer sequences are listed in Table 1.

### 4.12. Statistical Analysis

The results are presented as the mean ± standard deviation. Numerical outcomes among different groups were statistically analyzed using one-way analysis of variance (ANOVA) with post hoc verification through Dunnet’s multiple comparison test, conducted using GraphPad Prism (version 5.0; GraphPad Software, Inc., San Diego, CA 92130, USA). Significance was determined at * *p* < 0.05, ** *p* < 0.01, and *** *p* < 0.001. LEfSe of the gut microbiota was conducted based on the Kruskal–Wallis and Wilcoxon test results.

## 5. Conclusions

This study provides compelling evidence that oral administration of SSO can significantly promote hair growth. The enhancement of hair follicle proliferation, modulation of the expression of growth factors, and alterations in the gut microbiota composition collectively contribute to the observed hair growth-promoting effects. In addition, a new group of gut microbes that may interfere with the promotion of hair growth were identified. The microbes exhibiting a negative correlation between the promotion of hair growth and specific altered gut microbial groups belonged to *Peptostreptococcaceae*, *Criibacterium*, and *Intestinibacillus*. These findings pave the way for further research into the therapeutic potential of SSO and its application in treating hair loss conditions. Future studies should focus on elucidating the specific active constituents of SSO and their precise mechanisms of action, as well as on exploring their effects in human clinical trials.

## Figures and Tables

**Table 1:** Oligonucleotide sequencings.

Primer Sequence	Target Gene
5′-GTC TCC TCT GAC TTC AAC AGC G-3′	GAPDH-F
5′-ACC ACC CTG TTG CTG TAG CCA A-3′	GAPDH-R
5′-CTC TTC AGT TCG TGT GTG GAG AC-3′	IGF-1-F
5′-CAG CCT CCT TAG ATC ACA GCT C-3′	IGF-1-R
5′-TGC GAT GCC AAG CAG TCT GTG A-3′	EGF-F
5′-GCA TAG CCC AAT CTG AGA ACC AC-3′	EGF-R
5′-TGG CAA GCA CTA CCT GGA TCA G-3′	ERK-1-F
5′-GCA GAG ACT GTA GGT AGT TTC GG-3′	ERK-1-R
5′-TAC CTG AAC CCG TGT TGC TCT C-3′	TGF- β1-F
5′-GTT GCT GAG GTA TCG CCA GGA A-3′	TGF- β1-R
5′-AAG AAG CGT GCT TTG GAT GCG G-3′	TGF- β2-F
5′-ATG CTC CAG CAC AGA AGT TGG C-3′	TGF- β2-R
5′-TTG CCT TGC TGC TCT ACC TCC A-3′	VEGF-F
5′-GAT GGC AGT AGC TGC GCT GAT A-3′	VEGF-R
5′-GGT ATT CCA GAA GAA CCA CCT TG-3′	DKK-1-F
5′-CTT GGA CCA GAA GTG TCT AGC AC-3′	DKK-1-R
5′-TGT TCT GTC GCA CCC AGT GGT A-3′	FGF7-F
5′-TTC CAA CTG CCA CGG TCC TGA T-3′	FGF7-R
5′-TGA GAA GAA CGG GAA GGT CAG C-3′	FGF10-F
5′-TGG CTT TGA CGG CAA CAA CTC C-3′	FGF10-R
5′-GAG CGT TAC CAG AAC CTG TCT C-3′	P38-F
5′-AGT AAC CGC AGT TCT CTG TAG GT-3′	P38-R
5′-CAC AAG CAG AGT GCT GAA GGT G-3′	β-catenin-F
5′-GAT TCC TGA GAG TCC AAA GAC AG-3′	β-catenin-R

## Data Availability

Data will be made available on request.
